# The Association Between Cardiac Illness-Related Distress and Partner Support: The Moderating Role of Dyadic Coping

**DOI:** 10.3389/fpsyg.2021.624095

**Published:** 2021-02-17

**Authors:** Giada Rapelli, Silvia Donato, Ariela Francesca Pagani, Miriam Parise, Raffaella Iafrate, Giada Pietrabissa, Emanuele Maria Giusti, Gianluca Castelnuovo, Anna Bertoni

**Affiliations:** ^1^Department of Psychology, Family Studies and Research University Centre, Università Cattolica del Sacro Cuore, Milan, Italy; ^2^Psychology Research Laboratory, Istituto Auxologico Italiano, IRCCS, Milan, Italy; ^3^Department of Psychology, Università Cattolica del Sacro Cuore, Milan, Italy

**Keywords:** couple distress, dyadic coping, patient engagement, cardiac illness, partner support

## Abstract

Managing cardiac illness is not easy because it dramatically disrupts people’s daily life and both the patient and his/her spouse are at risk for experiencing distress, which, in turn, may affect the support provided by the partner as caregiver. The partner, in fact, is the main source of support, but his/her support may sometimes be inadequate. In addition, dyadic coping (i.e., the way partners cope together against stress and support each other in times of difficulty) could likely be a moderating factor. The main aim of the present study was to examine the role that dyadic coping (DC, in terms of positive, negative, and common dyadic coping responses) plays in moderating the link between patient and partner cardiac illness-related distress (in terms of anxiety and depression) and partner support (in terms of overprotection, hostility, and partner support for patient engagement). The study included 100 married couples faced with cardiac illness who completed a self-report questionnaire. We analyzed our data in PROCESS using multiple regressions in order to assess the moderating effects of DC responses in the relationship between the couple’s cardiac illness-related distress and partner support. With regard to patient distress, results showed that higher levels of patient anxiety and depression were linked with ineffective partner support (i.e., overprotection and hostility). With regard to partner distress, higher levels of partner depression were linked with hostility; higher levels of partner depression and anxiety were associated with less partner support for patient engagement. Moreover, the association between distress and partner support was moderated by the quality of DC. In particular, low positive DC represented a risk factor for both the patient and the partner during a cardiac illness, as low positive DC exacerbated the link between patient and partner distress and less effective partner support styles. Also, higher levels of negative DC were risky for couples: The association between distress and less adequate partner supportive behaviors was stronger in the case of higher negative DC. These results imply a need for psychosocial interventions for couples in cardiac illness, especially for couples lacking relational competences, such as positive dyadic coping.

## Introduction

Cardiac illness is a stressful situation because it disrupts daily life and demands many lifestyle changes (e.g., diet, physical activity, smoking and alcohol consumption, medical check-ups, prescription drug compliance, etc.). Evidence supports the view that cardiac patients suffer from stress in managing their clinical condition (Jackson et al., 2018). Still, illness management does not happen in isolation, and successfully coping with a cardiac disease significantly depends on the individual’s perceived social support, particularly that of the partner (Donato et al., 2009; Iafrate et al., 2009; Rapelli et al., 2020c). The partner usually provides emotional and practical support to the cardiac patient by constantly monitoring the patient’s medication adherence, making appointments, accompanying the partner to the regular medical visits, and detecting signs of cardiac symptoms. The partner, moreover, is the main person responsible for the patient’s low-salt diet, takes charge of the tasks that previously were done with or by the patient, and contributes actively to making decisions on health care (Tulloch et al., 2015). According to Bertoni et al. (2015), when the partner is able to provide adequate support (i.e., balancing emotional and practical support, involving the patient in discussions, not substituting for the patient, but reinforcing the patient’s autonomous capacities), the patient is more engaged in his/her treatment with benefits in terms of psychological well-being (see, for a review, Bertoni et al., 2017), disease management, and quality of life (Greene et al., 2015; Shortell et al., 2017). The cardiac event has significant implications for both the patient and his/her partner after the diagnosis and during the recovery. In fact, following a cardiac event, both the patient and his/her spouse are at risk of experiencing distress and face a number of challenges, including the fear surrounding the patient’s health and illness progression, the novelty and unpredictability of the cardiac event, disruption of goals, caregiving demands, and decreased perceived control over the patient’s illness (Leigh et al., 2014). The support provided by the partner, however, may often be inadequate because of his/her burden and because the caregiver may not know how to effectively support the patient (Dekel et al., 2014; Bertoni et al., 2015; George-Levi et al., 2017). The literature on cardiac patients has actually highlighted that the caregiver may implement overprotective or hostile support styles, both of which are associated with worse patient health outcomes. In particular, an overprotective partner underestimates the patient’s capabilities, resulting in unnecessary help, excessive praise for accomplishments, or attempts to restrict activities, thereby resulting in worse outcomes for the patient (Bertoni et al., 2020), such as decreased quality of life and self-efficacy (Joekes et al., 2007; Zniva et al., 2017). Instead, partners’ hostile behaviors are not just unskillful, but openly unsupportive and characterized by criticism, coldness, and blame (Fiske et al., 1991). Hostility is associated with decreased patient engagement in his/her care (Rapelli et al., 2020a), increased psychological distress, and higher risk of relapses (Fiske et al., 1991). Evidence exists for the partner’s (un)supportive behaviors to be associated with (low) patient well-being and (low) self-efficacy, but whether and how patients’ and partners’ distress is associated with specific types of support has not yet been clarified. In addition, research is needed on factors that can reduce or exacerbate the negative interplay between the patients’ and partners’ cardiac illness-related distress and the partner’s unsupportive behaviors.

Recent studies of stress and coping that account for the importance of social relationships in the coping process have increasingly emphasized a dyadic perspective on illness management (Bertoni et al., 2015; Donato and Bertoni, 2018; Rentscher, 2019). In couples, mutual coping processes with external stressors, such as an illness, are covered by Bodenmann’s concept of dyadic coping (1997; 2005), that is, the process through which partners cope together, as a couple, with daily stressors. In fact, when one partner’s individual resources are insufficient for coping with a stressor, he/she may share the stressful situation with the partner, who then interprets the stress signals and responds to the shared information with a behavioral response that can be either positive or negative (Bodenmann, 2005). Bodenmann (2005) distinguished three forms of dyadic coping: Positive dyadic coping, which refers to one partner’s attempts to assist the other’s coping efforts, including delegated dyadic coping (one partner asks the other to take over certain tasks and duties in an effort to reduce his or her stress experienced in the situation); negative dyadic coping that is composed of superficial, ambivalent, or hostile reactions to the partner’s stress; and common dyadic coping, in which both partners participate in the coping process, more or less symmetrically (e.g., through shared problem solving or mutual encouragement). Abundant research has found that positive and common dyadic coping are associated with lower levels of stress and higher levels of couple satisfaction, while the opposite was found for negative dyadic coping (Hilpert et al., 2016; Parise et al., 2019). Good dyadic coping competences, therefore, should protect the partner against the negative effects of (one’s own and the patient’s) distress on his/her support behaviors, in at least three ways. First, couples showing good dyadic coping skills should be able to better cope with the stress caused by the illness and, therefore, should be less affected by its negative impact. Second, dyadic coping is generally associated with better relationship quality as it is an indicator of how much the partners jointly commit to each other’s relationship satisfaction, quality of life, and mutual well-being (Bertoni et al., 2018). Therefore, partners with a better relationship quality and higher relationship satisfaction should present more benevolent interpretations of the patient’s distress and negative behaviors and rely less than dissatisfied partners do on self-defensive reactions (Bradbury and Fincham, 1990). Finally, partners with good dyadic coping skills are especially able to appraise the illness as a couple, rather than individual, problem (Falconier and Kuhn, 2019); consequently, they are more prone to respond with positive behaviors to cope with an illness that is *not only “yours,” but also “ours.”* No studies, however, have examined the potential moderating effects of dyadic coping in the link between (patient’s and partner’s) cardiac illness-related distress and the quality of partner support in the context of cardiac illness.

**FIGURE 1:**
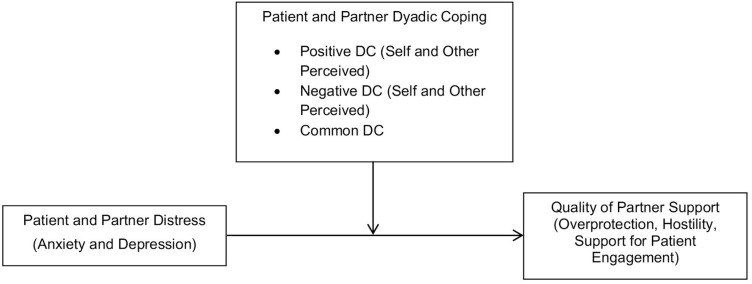
The moderation model used in this study. DC = Dyadic Coping.

Given the serious stress experienced by both patients and partners when facing cardiac illness, the crucial role played by the quality of partner support for the patient’s physical and psychological outcomes, and the potential for dyadic coping to function as a protective factor in this context, the main aim of the present study was to examine the role that dyadic coping plays in moderating the relation between patient’s and partner’s cardiac illness-related distress and partner support in 100 married couples faced with cardiac illness. This is the first study, to our knowledge, to analyze the above associations and to do so within a dyadic framework, which may help gain a deeper understanding of the role of the quality of interpersonal relationships in shaping cardiac disease management processes. In particular, positive, negative, and common dyadic coping were examined as moderators of the links between patient’s and partner’s distress (in terms of anxiety and depression) and the quality of partner support (Figure 1). In addition, the quality of partner support was assessed in terms of three (un)supportive behaviors already investigated in the cardiac population: Hostility (openly unsupportive behaviors; Coyne and Smith, 1994; Rapelli et al., 2020a), overprotection (well-intended, but unskillful support; Vilchinsky et al., 2010), and support for patient engagement (positive and skillful form of partner support aimed at increasing the patient’s autonomous skills in treatment; Bertoni et al., 2015).

In light of the literature reviewed above, we tested the following hypotheses:

**Hypothesis 1:** Patient cardiac illness-related distress (Hp1a) and partner cardiac illness-related distress (Hp1b) will be positively associated with partner hostility and partner overprotection and negatively associated with partner support for patient engagement;
**Hypothesis 2:** These associations will be stronger for patients (Hp2a) and partners (Hp2b) who perceive themselves and/or their partners to display low positive dyadic coping, high negative dyadic coping, and low common dyadic coping.

## Materials and Methods

### Participants and Procedure

One hundred and fifty patients were originally recruited within a larger research project on cardiovascular patients’ well-being: One hundred thirty-three of them were in a committed couple relationship. Only couples in which both patients and partners completed the questionnaire were selected for the current study, which resulted in a final sample composed of 100 heterosexual couples.

The socio-demographic characteristics of the sample are shown in Table 1. Patients (*N* = 100; 83% male) ranged in age from 34 to 85 years (*M* = 62.97, *SD* = 11.25); partners (*N* = 100; 83% female) were slightly younger on average, ranging in age from 31 to 87 years (*M* = 59.85; *SD* = 11.36). The couples were married or in a committed relationship for 3 to 60 years (*M* = 36.99; *SD* = 13.45). The main diseases for which patients were hospitalized were ischemic heart disease including angina pectoris and acute coronary syndrome (ST-segment elevation myocardial infarction–STEMI; non-ST-segment elevation myocardial infarction–NSTEMI) (75%), and acute heart failure (25%).

**TABLE 1:** Socio-demographic characteristics of couples (*N* = 100).

	Patients			Partners		
Variable	*M*	*SD*	Range	*M*	*SD*	Range
Age	59.85	11.36	31–87	60.50	11.30	31–87
Years of Education	14.1	2.9	5–20	14.0	2.9	5–20
Relationship Duration (years)	36.99	13.45	3–60	36.99	13.45	3–60
Presence of Children (%)	88.6					
First Marriage (%)	92.6			89.4		
Male (%)	83			17		
Employed (%)	51.0			52.3		

Participants were contacted and interviewed during the patient’s hospitalization for an acute cardiac event. A set of two questionnaires (one for the patient and one for the partner) was administered 2 days before discharge. Signed informed consent was obtained from all participants. Criteria for study inclusion were as follows: (1) Admission for acute cardiac illness (e.g., Ischemic heart diseases like myocardial infarction and acute coronary syndrome); (2) no mental disability, assessed with a short version of the Mini Mental State Examination (MMSE); and (3) ability to understand Italian and complete the questionnaire autonomously. The Psychology Research Ethic Committee of the Institution approved the study (cod. 37-18).

### Measures

The description of measures and internal consistency reliability coefficients (Cronbach’s alpha) for patients and partners are shown in Table 2.

**TABLE 2:** Measures and alpha’s coefficients.

Patient		Partner
α	Construct and scale’s characteristics	α
Individual Functioning		
Anxiety Self-Perceived: 0.81	Cardiac Illness-Related Distress Hopkins Symptom Checklist-25 (HSCL-25) (Mattsson et al., 1969) Clinical cutoff score for Anxiety and Depression = 1.70 Range = 1–4	Anxiety Self-Perceived: 0.83
Depression Self-Perceived: 0.82		Depression Self-Perceived: 0.87
Relational Functioning		
N/A	Partner Overprotection Michigan Family Heart Questionnaire (Fiske et al., 1991) Range = 1–5	Self-Perceived: 0.69
N/A	Partner Hostility Michigan Family Heart Questionnaire (Fiske et al., 1991) Range = 1–5	Self-Perceived: 0.66
N/A	Partner Support for Patient Engagement (*ad hoc*) Range = 1–5	Self-Perceived: 0.63
Positive DC Self-Perceived: 0.89	Dyadic Coping (DC) (Dyadic Coping Inventory; DCI) (Bodenmann, 1997) Range = 1–5	Positive DC Self-Perceived: 0.77
Positive DC Other-Perceived: 0.72		Positive DC Other-Perceived: 0.89
Negative DC Self-Perceived: 0.58		Negative DC Self-Perceived: 0.69
Negative DC Other-Perceived: 0.83		Negative DC Other-Perceived: 0.57
Common DC Self-Perceived: 0.75		Common DC Self-Perceived: 0.86

#### Cardiac Illness-Related Distress

Cardiac illness-related distress of both the patient and the partner was measured by a 25-item version of the Hopkins Symptom Checklist (HSCL-25; Mattsson et al., 1969). The scale consisted of 25 items measuring symptoms of anxiety, depression, and somatization. Both patients and partners were asked to rate the symptoms they experienced during the past week as ranging from 1 = *never* to 4 = *often* [e.g., *Item* # 1 for anxiety: *(In the past week, to what extent did you worry or stress for this symptom*…*) “Suddenly scared for no reason.”;* Item # 25 for depression: *(In the past week, to what extent did you worry or stress for this symptom*…*) “Difficulty in falling asleep and in sleeping?”*]. The total score of the subscales (anxiety and depression) are computed by averaging the items: Higher scores indicated more psychological distress. The cut-off clinical score was set at 1.70, according to the validation study (Mattsson et al., 1969).

#### Partner Hostility

The partner’s hostile attitude toward the patient was measured by the Spouse Hostility Scale from the Michigan Family Heart Questionnaire (Fiske et al., 1991). It consisted of five items which were included in the questionnaire filled in by the partner (e.g., Item # 5: *“My spouse doesn’t try hard enough to help himself/herself.”*). All responses were coded on a 5-point Likert-type scale ranging from 1 = *never* to 5 = *very often*. The total score of the scale was computed by averaging the five items: A higher score indicated a higher level of hostility.

#### Partner Overprotection

The partner unrequired protection and interference with the patient’s behaviors and decisions was measured by the Spouse Overprotection Scale from the Michigan Family Heart Questionnaire (Fiske et al., 1991). It consisted of four items which were included in the questionnaire filled in by the partner (e.g., Item # 4: *“I find myself stepping in and doing things that my spouse can do for himself.”*). All responses were coded on a 5-point Likert-type scale ranging from 1 = *never* to 5 = *very often*. The total score of the scale was computed by averaging the five items: A higher score indicated a higher level of overprotection.

#### Partner Support for Patient Engagement

The partner’s supportive behaviors aimed at promoting the patient’s active engagement into his/her treatment was measured with 11 *hoc* items on a 5-point Likert-type scale ranging from 1 = *strongly disagree* to 5 = *strongly agree* (e.g., Item # 11: *“I help my partner to recognize when he/she needs medical care and when he/she can manage the problem on his/her own.”*). The total score was created by averaging the items after reverse coding negatively keyed items: A higher score indicated a higher level of support for patient activation by the partner.

#### Patient and Partner Dyadic Coping

Dyadic coping of both the patient and the partner was measured with the Italian version of the Dyadic Coping Inventory (Fragebogen zur Erfassung des Dyadischen Copings als Tendenz FDCT-N; Bodenmann, 1997; Donato et al., 2009). This 41-item questionnaire measures the propensity of each partner to offer help, emotional support, and empathy in response to the other’s expression of stress together with the couple’s joint attempts to cope with common stressors. The scale considers the three forms of dyadic coping: Positive (e.g., *“My partner is on my side and tells me that he/she knows how it feels to be stressed and that he/she cares about me.”*), negative (e.g., *“My partner helps me, but does so unwillingly and unmotivated.”*), and common (e.g., *“We try to cope with the problem together and search for practical solutions.”*). For positive and negative dyadic coping, we considered both self-perceptions (from now on “dyadic coping self-perceived”) (e.g., *“When my partner is stressed, I communicate my understanding to him/her.”*) and the perceptions of the other (from now on “dyadic coping other-perceived”) (e.g., *“When I’m stressed, my partner gives me the feeling that he/she understands me.”*). The items were administered on a 5-point Likert-type scale from 1 = *never* to 5 = *very often*.

### Statistical Analyses

Descriptive statistics were obtained from the sample in terms of socio-demographic data. Pearson’s correlations were used to calculate the relationship between study variables. In order to test for differences between patients and partners on study variables, paired sample *t*-tests were calculated.

To examine the moderating effects of partners’ dyadic coping responses (moderators) in the link between patients’ and spouses’ distress (independent variables) and their partners’ support (dependent variable), we used PROCESS, a freely available computational tool for SPSS and SAS developed by Hayes (2017). To examine moderation effects in this study, we performed the analyses corresponding to PROCESS Model 1. A moderated model was tested in which patient and partner distress in terms of anxiety and depression were hypothesized to be associated with the quality of partner support in terms of overprotection, hostility, and support for patient engagement, as well as the moderating role of dyadic coping responses (positive, negative, and common) in these associations. Prior to model analyses, all predictors and moderators were mean-centered to reduce collinearity between the interaction term and its constituents (Aiken et al., 1991). Regression analyses were conducted in which coefficients were bootstrapped using 5,000 bootstrap samples. The coefficients were tested for statistical significance by means of the percentile confidence intervals, and a significant effect is said to occur if the 95% confidence interval excluded 0.

## Results

### Descriptive Statistics

Table 3 shows the means and correlations for the selected psychological variables. Anxiety was high and above the clinical cut-off score (1.70) for both patients (*M* = 1.72; *SD* = 0.56) and their partners (*M* = 1.83; *SD* = 0.58); depression was lower than the clinical cut-off score (1.70) (Patients: *M* = 1.66; *SD* = 0.51; Partners: *M* = 1.69; *SD* = 0.56). Of the three support styles, hostility and overprotection were moderate compared to the scale range (Hostility: *M* = 2.14; *SD* = 0.75; Overprotection: *M* = 2.85; *SD* = 0.86), partner support for patient engagement was high (*M* = 3.79; *SD* = 0.66) compared to the scale range. Positive dyadic coping self-perceived (Patients: *M* = 3.67; *SD* = 0.68; Partners: *M* = 3.69; *SD* = 0.63) and other-perceived (Patients: *M* = 3.88; *SD* = 0.79; Partners: *M* = 3.37; *SD* = 0.88) were high. Furthermore, partners reported receiving significantly less positive dyadic coping responses from the other than patients did [*t*(95) = 4.12; *p* = 0.008]. Negative dyadic coping self-perceived (Patients: *M* = 1.83; *SD* = 0.82; Partners: *M* = 1.96; *SD* = 0.95) and other-perceived (Patients: *M* = 1.88; *SD* = 0.69; Partners: *M* = 1.95; *SD* = 0.69) were low. Common dyadic coping was high (Patients: *M* = 3.47; *SD* = 0.76; Partners: *M* = 3.40; *SD* = 0.89).

**TABLE 3:** Means and intercorrelations among study variables.

Variables	1	2	3	4	5	6	7	8	9	10
1. Anxiety	–	0.71**	0.14	0.16	−0.19*	−0.03	−0.07	−0.01	0.03	−0.01
2. Depression	0.72**	–	0.08	0.28**	−0.20*	−0.02	−0.06	0.08	0.13	0.04
3. Partner Overprotection	0.24**	0.14	–	0.42**	−0.01	0.02	0.28**	0.01	−16	0.12
4. Partner Hostility	0.28**	0.21*	0.42**	–	−0.23*	−0.03	−0.02	0.23*	0.19	0.02
5. Partner Support to Patient Engagement	0.06	−0.19*	−0.01	−0.23*	–	0.42**	0.40**	−0.47**	−0.49**	0.33**
6. Positive DC (self-perceived)	−0.01	−0.01	0.31*	−0.15	0.25*	–	0.46**	−0.20*	−0.23*	0.60**
7. Positive DC (other-perceived)	−0.01	0.04	−0.01	−0.30**	0.18	0.34**	–	−0.26**	−0.34**	0.59**
8. Negative DC (self-perceived)	0.14	0.11	−0.02	0.43**	−0.29**	−0.10	−0.11	–	−53**	−0.32**
9. Negative DC (other-perceived)	0.23*	0.08	−0.08	0.36**	−0.25*	−0.19	−0.03	0.51**	–	−0.31
10. Common DC	0.12	0.11	0.04	−0.21*	0.11	0.50**	0.45**	−0.01	−0.13	–
Patients M (SD)	1.72 (0.56)	1.66 (0.51)	N/A	N/A	N/A	3.67 (0.68)	3.88 (0.79)	1.83 (0.82)	1.88 (0.69)	3.47 (0.76)
Partners M (SD)	1.83 (0.58)	1.69 (0.56)	2.85 (0.86)	2.14 (0.75)	3.79 (0.66)	3.69 (0.63)	3.37 (0.88)	1.96 (0.95)	1.95 0.69	3.40 (0.89)
*t*	−0.51	−0.47	N/A	N/A	N/A	0.72	4.12**	−1.03	−0.76	0.99

N = 100. Means and standard deviations of study variables for patients and partners are reported separately. Correlations for patients are above the diagonal; correlations for partners are below the diagonal. DC = Dyadic Coping *p < 0.05; **p < 0.01.

Table 3 also shows the correlation analysis among study variables. For patients, anxiety and depression were negatively correlated with partner support for patient engagement and hostility showed a weak positive association with depression. The associations between the support styles and dyadic coping were low to moderate in size.

For partners, anxiety was weakly and positively correlated with overprotection and hostility; depression was weakly and positively correlated with hostility and weakly and negatively correlated with partner support for patient engagement. The associations between the support styles and dyadic coping were low to moderate.

### Testing Moderating Effects

To test whether dyadic coping (i.e., self and other-perceived positive dyadic coping, self and other-perceived negative dyadic coping, and common dyadic coping) moderated the association between the patient’s and the partner’s cardiac illness-related distress and the partner’s support quality (i.e., overprotection, hostility, and partner support for patient engagement), we conducted several hierarchical regression analyses. To test whether these effects varied significantly across the levels of the moderators, the differences in the effects for high and low levels of the moderator were computed and tested for significance by determining the bootstrapped confidence limits of the difference. As suggested by Aiken et al. (1991), low and high levels of the moderators were defined as minus one standard deviation and plus one standard deviation of the moderators, respectively. The results for patients and for partners were presented separately. We reported only significant interaction effects.

#### Results for Patients’ Distress (Hp1a and Hp2a)

##### Partner Hostility

**FIGURE 2:**
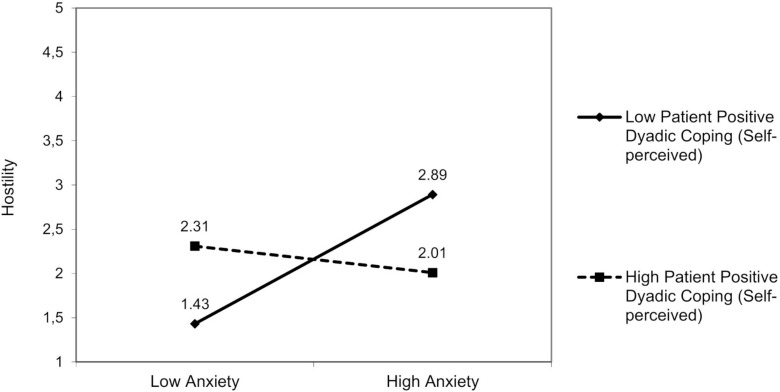
The interactive effect of patient anxiety and patient positive dyadic coping self-perceived on partner hostility.

A significant interaction effect between patient anxiety and patient positive dyadic coping (self-perceived) was found on partner hostility [*F*(3,92) = 2.81, *p* = 0.04]: Patient positive dyadic coping (self-perceived) moderated the effect of patient anxiety β = −0.22; 95% bootstrap CI (−0.42, −0.02)] on partner hostility (Figure 2). Patient anxiety was positively associated with partner hostility only when patients reported low positive dyadic coping, Δ*R^2^* = 0.05, Δ*F*(1,92) = 2.16, *p* = 0.04. For patient anxiety, we did not find any other moderating effects of dyadic coping.

**FIGURE 3:**
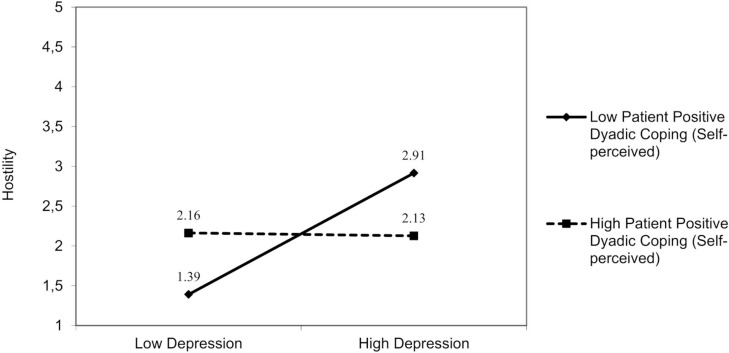
The interactive effect of patient depression and patient positive dyadic coping self-perceived on partner hostility.

**FIGURE 4:**
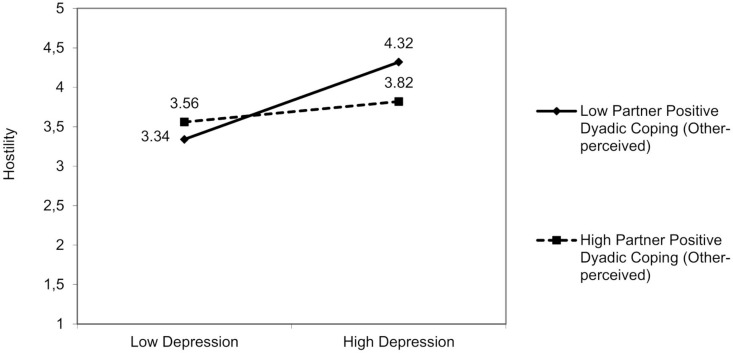
The interactive effect of patient depression and patient positive dyadic coping other-perceived on partner hostility.

For patient depression, a significant interaction effect, resulting from patient depression and patient positive dyadic coping (both self and other-perceived), was found on partner hostility. The first model was significant [*F*(3,94) = 5.32, *p* = 0.01]: Patient positive dyadic coping (self-perceived) moderated the effect of depression [*β* = −0.25; 95% bootstrap CI (−0.42, −0.08)] on partner hostility (Figure 3), Δ*R*^2^ = 0.08, Δ*F*(1,94) = 3.05, *p* = 0.01. In addition, in the second model [*F*(3,93) = 3.74, *p* = 0.01], patient positive dyadic coping (other perceived) moderated the effect of depression [*β* = −0.18; 95% bootstrap CI (−0.01, −0.37)] on partner hostility (Figure 4), Δ*R*^2^ = 0.03, Δ*F*(1,94) = 3.05, *p* = 0.01. The patient depression was positively associated with partner hostility only when patients reported engaging in low positive dyadic coping and perceived their partner as adopting low positive dyadic coping.

##### Partner Overprotection

**FIGURE 5:**
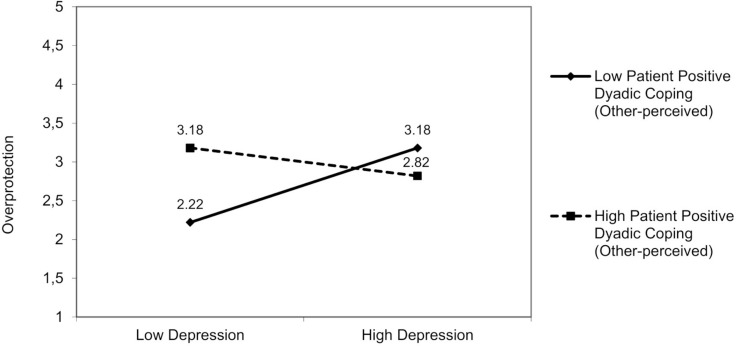
The interactive effect of patient depression and patient positive dyadic coping other-perceived on partner overprotection.

**FIGURE 6:**
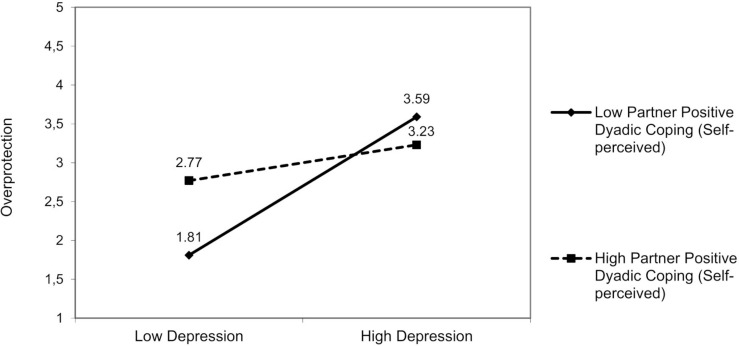
The interactive effect of patient depression and partner positive dyadic coping self-perceived on partner overprotection.

No interactions involved patient anxiety. A significant interaction effect was found for patient depression and patient positive dyadic coping (other-perceived) on overprotection [*F*(3,94) = 4.96, *p* = 0.01]. The patient’s depression was negatively associated with overprotection, but only in those patients who perceived that their partners had adopted low positive dyadic coping [*β* = −0.15; 95% bootstrap CI (−0.31, −0.02); Figure 5], Δ*R*^2^ = 0.04, Δ*F*(1,94) = 0.66, *p* = 0.04. Furthermore, we found an interaction between patient depression and partner-reported positive dyadic coping (self-perceived) on overprotection [*F*(3,93) = 5.15, *p* = 0.01]. The patient’s depression was negatively associated with overprotection, but only in patients whose partners reported low positive dyadic coping [*β* = −0.15; 95% bootstrap CI (−0.30, −0.01); Figure 6], Δ*R*^2^ = 0.04, Δ*F*(1,94) = 1.37, *p* = 0.04.

##### Partner Support for Patient Engagement

For partner support for patient engagement, we did not find any moderating effects of dyadic coping.

#### Results for Partners’ Distress (Hp1b and Hp2b)

##### Partner Hostility

**FIGURE 7:**
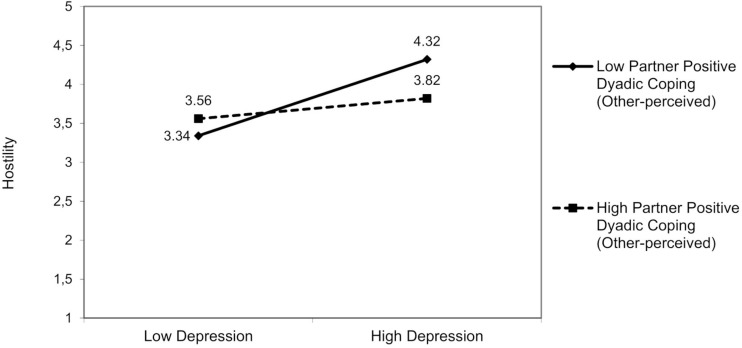
The interactive effect of partner depression and partner positive dyadic coping other-perceived on partner hostility.

No interactions involved partner anxiety. For partner depression, a significant interaction effect was found resulting from partner depression and partner positive dyadic coping (other-perceived) on hostility [*F*(3,94) = 6.72, *p* = 0.01]. The partner’s depression was positively associated with hostility, but only in those partners who perceived that the patient adopted low positive dyadic coping [*β* = −0.18; 95% bootstrap CI (−0.36, −0.01); Figure 7], Δ*R*^2^ = 0.04, Δ*F*(1,94) = 1.24, *p* = 0.03.

##### Partner Overprotection

For partner overprotection, we did not find any other moderating effects of dyadic coping.

##### Partner Support for Patient Engagement

**FIGURE 8:**
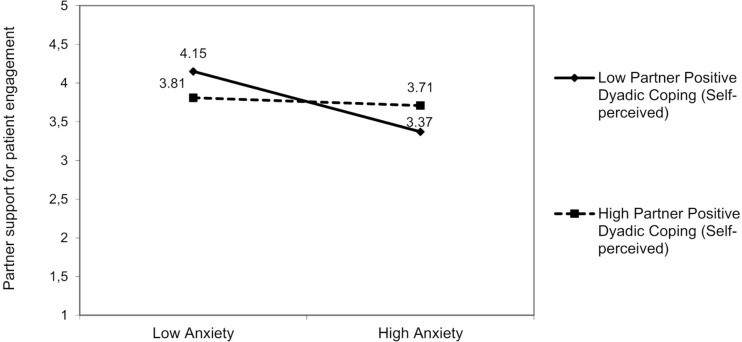
The interactive effect of partner anxiety and partner positive dyadic coping self-perceived on partner support for patient engagement.

**FIGURE 9:**
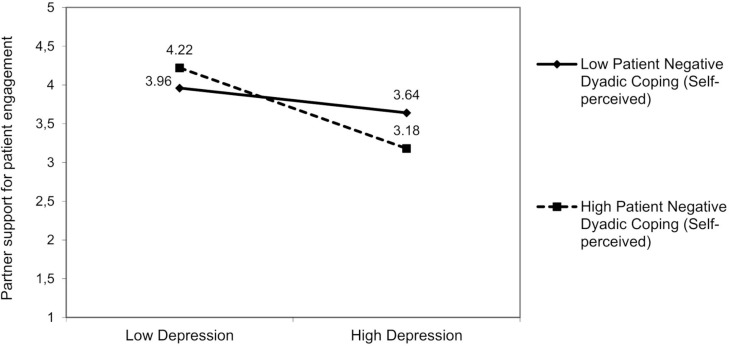
The interactive effect of partner depression and patient negative dyadic coping self-perceived on partner support for patient engagement.

There was a significant interaction effect resulting from partner anxiety and partner positive dyadic coping (self-perceived) on partner support for patient engagement [*F*(3,92) = 5.47, *p* = 0.01]: Partners’ positive dyadic coping (self-perceived) moderated the effect of their anxiety [*β* = −0.17; 95% bootstrap CI (−0.36, −0.01)] on partner support for patient engagement (Figure 8). The partner anxiety was negatively associated with partner support for patient engagement, but only in partners who reported low positive dyadic coping, Δ*R*^2^ = 0.04, Δ*F*(1,94) = 1.28, *p* = 0.04. For partner anxiety, we did not find any other moderating effects of dyadic coping. Furthermore, also patient-reported negative dyadic coping (other-perceived) moderated the link between partner depression and partner support for patient engagement [*F*(3,93) = 5.36, *p* = 0.01]. The partner’s depression was negatively associated with partner support for patient engagement, but only when patients reported that their partners adopted negative dyadic coping relatively often [*β* = −0.17; 95% bootstrap CI (−0.37, −0.01); Figure 9], Δ*R*^2^ = 0.03, Δ*F*(1,93) = 1.33, *p* = 0.04.

## Discussion

The present study examined whether the couple’s cardiac illness-related distress, measured separately for patients and their partners in terms of anxiety and depression, was associated with three types of partner support (overprotection, hostility, and support for patient engagement) and whether dyadic coping skills moderated this association. Although dyadic coping is highly predictive of relationship quality and stability (e.g., Donato et al., 2014, 2015; Falconier et al., 2015), which are factors considered as protective both for the cardiac patient in terms of survival (Coyne et al., 2001; Rohrbaugh et al., 2002) and for the caregiver’s psychological well-being (Dekel et al., 2014), no studies to date have considered dyadic coping as a moderator of the link between patient and partner distress and partner support.

Our hypotheses were that patient’s and partner’s cardiac illness-related distress would be associated with the quality of partner support. In fact, partner support may sometimes be detrimental and ineffective in the presence of patient and/or partner cardiac illness-related distress (George-Levi et al., 2016). Indeed, on the one hand, the partners are not experts in providing the care and may suddenly find themselves supporting the sick partner without knowing what to do, which could be difficult for them especially if the patient is anxious or depressed. The patient’s emotional distress could consequently aggravate the caregiver’s burden and cause inadequate support (Rapelli et al., 2020b). On the other hand, anxious or depressed partners may struggle to provide adequate support, because they themselves are challenged by the stress of the disease. This underlines that partner distress could have a negative impact on the quality of support provided and consequently on patient outcomes, as assumed by the literature (e.g., Franks et al., 2006; Bertoni et al., 2015; Rapelli et al., 2020a). Our results are in line with this scenario. In fact, higher levels of patient anxiety and depression were associated with higher ineffective partner support such as overprotection and hostility; higher levels of partner depression were linked with higher hostility, and higher levels of partner depression and anxiety were associated with less support for patient engagement. In addition, because our results found more significant patterns for patient cardiac illness-related distress than for partner cardiac illness-related distress, we could say that patient’s cardiac illness-related distress is more associated with unsupportive partner behaviors than partner’s psychological state. Moreover, we hypothesized that dyadic coping could work as a protective factor in the link between patients’ and partners’ distress and partner support. A distinction was made between positive, negative, and common dyadic coping (Bodenmann, 1997, 2005). In a recent review, Falconier and Kuhn (2019) documented that all positive dyadic coping strategies, including common dyadic coping, were significant positive predictors of individual outcomes and relationship satisfaction for both patients and partners, whereas all negative dyadic coping strategies were significant negative predictors. In particular, we expected positive and common dyadic coping to alleviate the effects of patients and partners’ distress on partner support, but negative dyadic coping to exacerbate it.

Results showed different interactive effects from dyadic coping, in particular, positive and negative forms of dyadic coping were significant moderators of the relationship between patient and partner cardiac illness-related distress and partner support. Our results suggest at least three reflections on the role of dyadic coping as a protective factor during an illness.

First, to better face the challenge of heart disease and stress, both the patient and the partner should show each other good dyadic skills, because the sharing of difficulties and the perception of the marital relationship as supportive and useful (Rusu et al., 2020) may increase the feeling of trust, intimacy, and reciprocity and decrease the negative impact of both partners’ stress on each other (Falconier and Kuhn, 2019).

Second, both self- and other-perceived dyadic coping moderated the link between partners’ cardiac illness-related distress and support, which means that the process by which cardiac illness-related distress was associated with support was truly relational. In addition, this finding suggests that not only the reported behaviors, but also the perceptions of partners’ responses are crucial for positive relationship exchanges, even during an illness. In particular, it is important for patients and their partners to reciprocate in showing some form of support for each other, whether instrumental or emotional, thereby restoring the balance within the relationship. In fact, not only should the caregiver support the patient in a one-way direction, but the amount of support the patient is able to give to the spouse is extremely important.

Indeed, perceived inequity and lack of reciprocity among partners was found to predict lower couple satisfaction (Iafrate et al., 2012a). In addition, the complementarity of dyadic coping efforts can be functional for the couple’s well-being (Revenson, 2003), especially in illness situations. In the present sample, the partner reported that the patient provided a significantly lower positive dyadic coping score than the one provided by the partner himself/herself, thereby suggesting a potential for perceived inequity. This result could be also explained by the fact that partners in our sample mostly comprised women and, according to the literature, women are more “relation-oriented” and more sensitive than men to the relationship aspects (Iafrate et al., 2012a).

Third, the moderating effect of dyadic coping was played in most interactions by positive dyadic coping; in particular, the effects of distress on unsupportive partner behaviors, such as hostile or overprotective styles, were particularly deleterious when they were combined with low self-perceived or other-perceived positive dyadic coping; conversely, high negative dyadic coping exacerbates the link among partner distress and lower support for patient engagement. Beyond the studies that detect the negative impact of negative dyadic coping (e.g., Gasbarrini et al., 2015; Falconier and Kuhn, 2019), the present study suggests that even low positive dyadic coping could have harmful effects, especially in a disease situation. This means that increasing the positive aspects of a marital relationship, such as the partners’ ability to understand each other, to look at stress from a different perspective, to encourage the partner, and help him/her concretely to solve the stressful problem, is crucial also during an illness. This is in line with recent literature showing the important role for couples of positive relational processes, for example, capitalization (Pagani et al., 2015, 2020; Donato et al., 2018).

Limitations of the present study should be noted. First, the cross-sectional nature of the design does not allow for inferences about the etiology of patient’s or partner’s cardiac illness-related distress, about whether distress drives perceptions of partner support or partner support drives distress, or about how distress may have coevolved with the partners’ functioning as a couple. In addition, the lack of a gender-balanced sample does not allow us to test gender differences in patients and partners. In fact, although heart disease has a higher incidence in the male population, a more balanced sample would help disentangle gender and role (patient and partner) effects. Moreover, our sample was mostly composed of stable couples and relatively limited in the age-range of participants: We could not test, therefore, whether interaction effects may differ as a function of relationship duration and partners’ age. Finally, we analyzed data through multiple regressions, but further studies based on structural equation modeling are needed to evaluate simultaneously multiple relationships among variables (e.g., Rusu et al., 2015). To our knowledge, however, this is the first study that investigates the relationship between distress, partner support, and the moderating role played by dyadic coping in a cardiac population; furthermore, it is a dyadic study; therefore, the perceptions of the distress and dyadic coping received and provided by both the patient and the partner are analyzed.

The present findings have also implications for interventions designed for couples facing cardiac illness. First, the involvement of partners in cardiac recovery programs recommended also as a best practice routine by the Italian Association for Cardiovascular Prevention, Rehabilitation, and Epidemiology (GICR-IACPR; Sommaruga et al., 2018). Secondly, the clinical importance of improving the dyadic coping skills in the couple, in accordance with Iafrate et al. (2012b), both in marital distress prevention programs and in marital therapy for couples facing a cardiac illness. In line with the literature (Falconier and Kuhn, 2019), in fact, strengthening positive dyadic coping and decreasing negative dyadic coping in a couple facing cardiac disease, beyond the effects on partners’ and couples’ well-being, could contribute to a more sustaining relationship, with consequent improvement of physical and psychological outcomes.

To conclude, the results of our study suggest the importance of including relational variables as moderators in the link between individual’s psychological state and the support provided by the partner. In fact, by distinguishing between the dyadic coping levels, it is possible to recognize individuals most at risk. In particular, in our study the patients and partners most at risk of receiving or implementing ineffective support for the patient seem to be those with high levels of distress combined with a low positive dyadic coping or high negative dyadic coping. This could suggest that a low positive dyadic coping and high negative dyadic coping exacerbate the association between patient and partner distress and ineffective partner support; consequently, in order to help the caregiver in his/her supportive role, it could be important to be engaged in a marital relation in which partners usually cope together with daily stress, show mutual empathy, encourage and help each other to put the problem in perspective, are committed to improve marital adjustment and well-being of the other, and can delegate to the other.

## Data Availability Statement

The raw data supporting the conclusions of this article will be made available by the authors, without undue reservation.

## Ethics Statement

The studies involving human participants were reviewed and approved by the Psychology Research Ethics Committee of Università Cattolica del Sacro Cuore di Milano. The patients/participants provided their written informed consent to participate in this study.

## Author Contributions

GP and SD contributed to the development of the theoretical framework, to the performance of the statistical analyses, to the analysis of the results, and to the writing of the manuscript. AP, MP, RI, GP, EG, and GC contributed to the development of the theoretical framework and to the writing of the manuscript. AB supervised the writing of the manuscript. All authors contributed to the article and approved the submitted version.

## Conflict of Interest

The authors declare that the research was conducted in the absence of any commercial or financial relationships that could be construed as a potential conflict of interest.
